# Peptide vaccine against chikungunya virus: immuno-informatics combined with molecular docking approach

**DOI:** 10.1186/s12967-018-1672-7

**Published:** 2018-10-27

**Authors:** Muhammad Tahir ul Qamar, Amna Bari, Muhammad Muzammal Adeel, Arooma Maryam, Usman Ali Ashfaq, Xiaoyong Du, Iqra Muneer, Hafiz Ishfaq Ahmad, Jia Wang

**Affiliations:** 1 Hubei Key Laboratory of Agricultural Bioinformatics, College of Informatics, Huazhong Agricultural University (HZAU), Wuhan, People’s Republic of China; 2 Department of Bioinformatics and Biotechnology, Government College University Faisalabad (GCUF), Faisalabad, Pakistan; 3 Department of Biosciences, COMSATS Institute of Information Technology (CIIT), Islamabad, Pakistan; 4 Key Lab of Animal Genetics, Breeding and Reproduction of Ministry Education, College of Animal Sciences & Technology, Huazhong Agricultural University, Wuhan, People’s Republic of China; 5 School of Life Sciences, University of Science and Technology of China, Hefei, People’s Republic of China

**Keywords:** Chikungunya virus (CHIKV), B cell and T cell epitopes, Vaccine, Computational approaches

## Abstract

**Background:**

Chikungunya virus (CHIKV), causes massive outbreaks of chikungunya infection in several regions of Asia, Africa and Central/South America. Being positive sense RNA virus, CHIKV replication within the host resulting in its genome mutation and led to difficulties in creation of vaccine, drugs and treatment strategies. Vector control strategy has been a gold standard to combat spreading of CHIKV infection, but to eradicate a species from the face of earth is not an easy task. Therefore, alongside vector control, there is a dire need to prevent the infection through vaccine as well as through antiviral strategies.

**Methods:**

This study was designed to find out conserved B cell and T cell epitopes of CHIKV structural proteins through immuno-informatics and computational approaches, which may play an important role in evoking the immune responses against CHIKV.

**Results:**

Several conserved cytotoxic T-lymphocyte epitopes, linear and conformational B cell epitopes were predicted for CHIKV structural polyprotein and their antigenicity was calculated. Among B-cell epitopes “PPFGAGRPGQFGDI” showed a high antigenicity score and it may be highly immunogenic. In case of T cell epitopes, MHC class I peptides ‘TAECKDKNL’ and MHC class II peptides ‘VRYKCNCGG’ were found extremely antigenic.

**Conclusion:**

The study led to the discovery of various epitopes, conserved among various strains belonging to different countries. The potential antigenic epitopes can be successfully utilized in designing novel vaccines for combating and eradication of CHIKV disease.

**Electronic supplementary material:**

The online version of this article (10.1186/s12967-018-1672-7) contains supplementary material, which is available to authorized users.

## Background

From mid 1950s till now, chikungunya fever is posing a substantial global health threat. The infection caused by an arthropod borne virus namely chikungunya virus (CHIKV), was initially reported in Africa and later on disease outbreaks specifically restricted to the tropical regions of South-East Asia, however West Pacific Islands were also documented. In Pakistan CHIKV was first disseminated in 2016 and affected 30,000 people in coastal region of Karachi. Of 30,000 cases, only 803 cases came into the knowledge of World Health Organization (WHO) [1].

Human epithelial, fibroblasts, macrophages and progenitor of muscle cells are primary targets of CHIKV. Chikungunya infection is characterized by an abrupt onset of headache, nausea, fatigue, muscles pain, crippling joint pains, high-grade fever and the prominent rashes. Wide variety of clinical appearances comprising myalgia, vomiting, muscular and joint pain has been observed in acute cases, while depression after several months of acute infection is considered the most lethal form of chikungunya infection. Severe acute arthritis is produced through the destruction of collagen which alters metabolism of connective tissues in cartilage [2–7]. Chronic symptoms include development of degenerative chronic lesions, loss of vision, sleep disorder and memory loss [2, 8].

Genome of CHIKV is comprised of single stranded RNA which is approximately 11.8 kb in size and has 11,990 base pairs. It has been classified as an alphavirus of *Togaviridae* family. It is predominantly spread by two mosquito borne vectors namely *Aedes aegypti* and *Aedes albopictus,* and that is why chikungunya fever and dengue infection shares analogous clinical manifestations [9]. Genome of CHIKV contains two open reading frames (ORF) and short non-translatable regions present on both 5′ and 3′ ends. Two ORF encode four non-structural (nsP1, nsP2, nsP3, nsP4) and structural (Capsid, 6K, E1 and p62) proteins. These unique virus encoded proteins are multifunctional and these are imperative to viral replication machinery [10]. Structural proteins are indispensable to fusion and entrance of virus into the host cell thus considered as important target for vaccine and antiviral drug development [11].

Till now, no licensed therapeutic or vaccine for the treatment of chikungunya infection is available in the market. Efforts for the discovery of antiviral drugs are at very early stages now. One of the successful approaches used for designing antiviral therapies is to target the non-structural proteins, so that multiplication of virus within the host can be controlled. But in CHIKV unlike other alphaviral proteins, non-structural proteins that make up active enzymatic complexes of its replication machinery are very difficult to target. Out of the four non-structural proteins, core polymerase subunits nsP4 is extremely challenging as it is inactive on its own [12]. The nsP2 enzyme which behaves as RNA helicase as well as protease enzyme is an extremely challenging target for inhibitor design. Unlike dengue and Hepatitis C proteases, cystein protease of CHKIV nsP2 bears a partially constricted substrate binding site which makes it difficult to exploit for the binding of protease blockers [13]. Likewise membrane association of nsP1 (RNA capping enzyme) has hampered the efforts of more specific cap methyltransferase or guanylyltransferase blockers [14]. Next comes to the list of structural proteins is nsP3, a replicase protein whose functions are still uncertain.

Currently, recombinant CHIKV vaccine candidates have been proposed by using adenovirus, vesicular stomatitis virus and measles virus vectors which are focused on expression of CHIKV structural genes to induce robust immune responses within the host. These vaccines protected mice from arthritis and helped in clearing viral load. But none of them has been evaluated in clinical trials [15–17]. Thus, the efforts for more potent and specific CHIKV therapeutic solution are still in its infancy. The current study is focused to find out conserved B cell and T cell epitopes of CHIKV structural polyprotein to be exploited for CHIKV vaccine discovery. In silico peptide prediction has been used in various studies to design therapeutic solutions against Zika virus and Hepatitis C Virus [10, 18, 19]. This study involves the application of computational approach to analyze the peculiar genomic properties of B cell and T cell epitopes on surface exposed antigenic protein which will induce a protective immunogenic response and helps lower the virus challenge.

## Methods

### Dataset collection and structural analysis

Primary sequence of the CHIKV structural polyprotein was obtained from the Uniprot (ID: Q1H8W5). Experimentally known crystal structure of CHIKV structural polyprotein was retrieved from Protein Data Bank (http://www.rcsb.org/pdb) (PDBId: 3N44). Protein sequence was analyzed for its physical and chemicals properties including half-life, GRAVY (Grand average of hydrophathicity), stability index, molecular weight and amino acid atomic composition through an online server Protparam [20]. Secondary structure of our CHIKV structural polyprotein was analyzed through PSIPRED [21].

### Prediction of B cell epitopes

Systematic strategy was used to design CHIKV structural polyprotein B and T cell epitopes. TMHMM was used for transmembrane topology prediction [22]. Freely available servers IEDB [23] and BCPREDS were recruited for B cell epitopes prediction [24]. Intracellular epitopes were excluded and only those epitopes were selected that were exposed on extracellular surface. Vaxijen 2.0 server was used for antigenicity analysis of selected epitopes [25]. Identification of B-cell epitopes was based on; antigenicity, flexibility, accessibility of surface, predictions of linear epitope and hydrophilicity [26]. Flexibility, accessibility of surface, isolation of linear epitope and hydrophilicity analysis were performed through Karplus and Schulz flexibility prediction tool, Emini surface accessibility prediction tool, Kolaskar and Tongaonkar antigenicity scale, Parker hydrophilicity prediction [26] and Bepipred linear epitope prediction algorithms. Prediction of beta turns in polyprotein was carried out by using Chou and Fasman beta turn prediction algorithm [27]. DiscoTope 2.0 server (http://www.cbs.dtu.dk/services/DiscoTope/) was used to model B cell epitopes from 3D structure of CHIKV structural polyprotein. All predicted epitopes were given in plain format while the 3D structure of structural polyprotein was given in PDB format.

### Prediction of T cell epitopes

By using two different online available tools, CTL epitopes of targeted protein were predicted. Major Histocompatibility Complex (MHC) class I and II were predicted by ProPred-1 (http://crdd.osdd.net/raghava/propred1/) and Propred tool (http://crdd.osdd.net/raghava/propred/), respectively. The predicted results from both these tools are quite significant because they use huge number of alleles of HLAs (human leukocyte antigens) during calculations. For class I allele’s prediction, the threshold value of Proteasome and immune-proteasome were set to 5%.

### Conservation analysis of selected epitopes

Sequences of CHIKV structural polyprotein, belonging to 23 different countries, were taken from Genbank and Uniprot databases. Multiple sequence alignment (MSA) was performed to find out the conservation of selected epitopes by using CLC work bench (https://www.qiagenbioinformatics.com/products/clc-main-workbench/). The aligned files (.aln) were further used to construct phylogenetic tree through MEGA7 software (https://www.megasoftware.net/). Epitopes were selected based on their conserved regions. Moreover, Immune epitope database and analysis resource (IEDB) was used to cross-check the conservation of the selected epitopes.

### Structure modeling and molecular docking

3-D structure of all the predicted peptides were modeled through PEPFOLD server at RPBS MOBYL portal [28, 29], while 3D structure of human HLA-B7 allele crystallized at a resolution of 1.7 Å was taken from Protein databank (PDB ID: 3VCL), was used for molecular docking purpose. Peptide models (antigenic determinants) were docked against their respective HLA-B7 allele through Molecular Operating Environment (MOE) tool to analyze their inhibitory potential. Protocol for molecular docking using MOE has already been reported in various studies involving the antiviral discovery against dengue non-structural proteases [30–32]. Docking protocol used in those studies involves removal of already bound peptide, protonation, energy minimization followed by removal of water molecules. Triangular matcher algorithm was applied as default peptide placement methods based on the receptor shape which quickly generates 1000 best poses of docked peptide without energy optimization. Energy estimation of the simulated poses was re-scored by applying London dG scoring function [33]. Functional form of London dG scoring function estimates ΔG of binding taking into account ligand flexibility, H-bonds, desolvation of each atom by applying equation given below:

$$ \Delta G = c + E_{flex} + \sum\limits_{h - bonds} {c_{HB} f_{HB} } + \sum\limits_{m - lig} {c_{M} f_{M} } + \sum\limits_{atoms\;i} {\Delta D_{i} } $$

where, c is average energy loss/gain due to receptor–ligand movement, *E*_*flex*_ measures entropic loss because of the conformational flexibility of the ligand, *c*_*HB*_ estimates H-bond maximum energy whereas H-bond *f*_*HB*_ accounts for geometric imperfections. In case of metals *c*_*M*_ and *f*_*M*_ measures metal ligation energy. While, ΔD calculates desolvation energy of each atom by integrating London dispersion forces over solvent space through the formula given below:

$$ \Delta D_{i} = c_{i} R_{i}^{3} \left\{ {\iiint\limits_{u \notin A \cup B} {\left| u \right|^{ - 6} du} - \iiint\limits_{u \notin B} {\left| u \right|^{ - 6} du}} \right\} $$

For each peptide, top 10 ranked poses of London dG were further minimized by Force field refinement algorithm. Protein peptide interactions were than analyzed through LigX tool of MOE. Pymol and UCSF Chimera tools were used to generate figures of docked complexes [34].

### Allergenicity assessment

Allergenicity analysis of predicted epitopes was done by Aller Hunter server (https://omictools.com/allerhunter-tool). This server compares query sequences of peptides against the database of already reported allergens to give significant results.

## Results

### Structural detail

The primary structure of the target sequence (Uniprot ID:Q1H8W5) was analyzed via Protparam. Physiochemical parameters of the CHIKV structural polyprotein computed through Protparam shows that protein is comprised of 1248 amino acid (aa) and have molecular mass of 138.31 kD. Theoretical iso-electric point (PI) of our subject protein was 8.90 which show its positive nature. Briefly, out of 1248 residues, 138aa were found as positively charged and 106 were calculated as negatively charged residues while Methionine (Met) was selected as an N-terminal residue in our subject structure. Protparam computed instability index, aliphatic index and GRAVY of target protein were 40.75, 74.93 and − 0.305 respectively.

Secondary as well 3D structure analysis of structural polyprotein through PSIPRED and UCSF Chimera respectively showed 50% beta sheet, 5% helixes and 45% loops (Additional file 1: Figure S1A). Crystal structure of structural polyprotein shown in Additional file 1: Figure S1B, C is a heteromer with chain A (blue) chain B (green) and chain F (red). Moreover, DiANNA 1.1 tool computed 24 disulfide (S–S) bond positions in target protein and assigned them score given in Additional file 1: Table S1. In the entire sequence, 49 cystine residues were calculated that are making 24 disulfide bonds at following sequence positions (106–416, 113–283, 128–287, 164–344, 269–741, 278–591, 286–887, 318–1068, 347–790, 353–714, 430–871, 450–1115, 478–550, 528–1080, 545–789, 716–1189, 721–781, 742–923, 783–903, 791–1242, 858–877, 872–1137, 905–1110, 1179–1185).

### Identification of B and T cell epitopes

Vaxijen 2.0 server confirmed the antigenicity value of target protein which was 0.5106. Prediction of transmembrane topology of the target protein was accomplished by TMHMM, showing five transmembrane helices that have range from 690–712, 733–755, 765–787, 794–816 and 1223–1245 sequence positions. Residue ranging from 1–689, 756–764 and 817–1222 sequence positions were exposed on the surface whereas residues from 713–732, 788–793 and 1246–1248 were buried inside the protein structure.

### Recognition of B cell epitopes

**Fig. 1:**
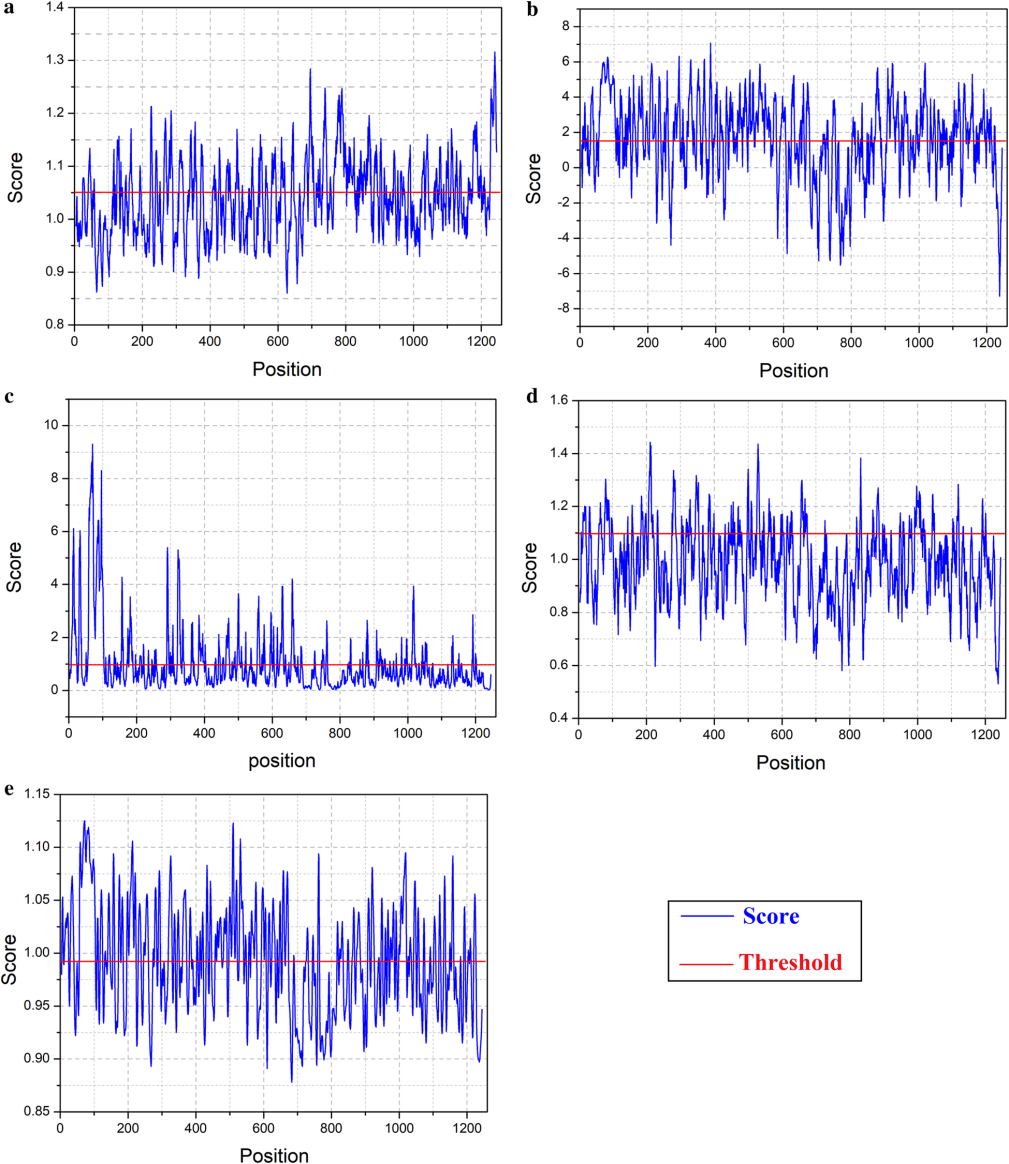
**a** Prediction of antigenic determinants using Kolaskar and Tongaonkar antigenicity scale, **b** hydrophilicity prediction using Parker hydrophilicity, **c** surface accessibility analyses using Emini surface accessibility scale, **d** beta turns analyses in structural polyprotein using Chou and Fasman beta turn prediction, **e** flexibility analyses using Karplus and Schulz flexibility scale

Potential B-cell epitopes have distinct characteristics that guide B cells to identify and trigger robust immune response against different viral infections. Primary sequence of target protein was scanned through BCPRED and different tools of the IEDB were used to predict B cell epitopes. From all the BCPRED predicted epitopes, only eight epitopes were observed to be antigenic in nature through Vaxigen computed score (Table 1). BCPRED has predicted B cell epitopes with 75% accuracy. Fourteen residues long epitopes are considered to be sufficient to induce protective immune response. Epitopes PPFGAGRPGQFGDI, IPTGAGKPGDSGRP, NYPASHTTLGVQDI, PFHHDPPVIGREKF, TSAPCTITGTMGHF, TPYELTPGATVPFL, PEGYYNWHHGAVQY and WLKERGASLQHTAP were predicted with 1, 0.999, 0.973, 0.97, 0.92, 0.858, 0.745 and 0.711 score respectively. In addition to this it is necessary to find out the surface accessibility of potential B-cell epitopes. Kolaskar and Tongaonkar antigenicity measuring tools analyzed the structural polyprotein for prediction of B-cell epitopes by computing their physicochemical properties of its amino acid and their profusion in known B cell epitopes. Kolaskar and Tongaonkar analysis of highly antigenic peptides is given in Fig. 1a. The threshold value of calculation was set at 1.041 while window size was kept 7. It predicted the antigenic propensity values of protein, which are 1.041 (average), 0.860 (minimum) and 1.316 (maximum). Hydrophilic regions of proteins are mostly exposed on the surface area, which potentially play an immense role in evoking immune response. BCPRED score and computed antigenicity results of Vaxigen clearly demonstrates that except the first peptide highlighted in italic in Table 1, all of our predicted peptides are part of extracellular region of transmembrane protein and have the power to maximize an immune response within the host during CHIKV infection. In addition to the above mentioned analysis, identification of B cell epitopes was done by Disco Top 2.0 server by analyzing 3-D structure of CHIKV structural polyprotein. Threshold range was kept at − 2.0 and a total of seven epitopes were computed as distinct surface exposed regions, shown in Table 2. In Kolaskar and Tongaonkar analysis the threshold value of calculation was set at 1.041 and window size was kept 7. It predicted the antigenic propensity values of protein in range of 0.860–1.316 shown in Fig. 1a. Hydrophilic regions of proteins were mostly exposed on the surface area, which potentially play an immense role in evoking immune response. Therefore, it is necessary to find out the surface accessibility of potential B-cell epitopes. For surface accessibility analysis Parker hydrophilicity and Emini surface accessibility prediction tools were used. Emini surface accessibility analyzing tool’s results are given in Additional file 1: Table S2, while graphical representations of the outputs of both tools shown in Fig. 1b, c, respectively. These peptides were part of extracellular region of transmembrane protein and have the power to maximize an immune response. Higher antigenicity score has suggested that these peptides can play a crucial role in initiation of immune response.

**Table 1:** B cell epitopes predicted through BCPRED

Serial no	Position	Peptide sequence	BCPRED score	Vaxigen score
1	*999*	*PPFGAGRPGQFGDI*	*1*	*1.663*
2	203	IPTGAGKPGDSGRP	0.999	1.1666
3	1198	NYPASHTTLGVQDI	0.973	1.2162
4	453	PFHHDPPVIGREKF	0.97	0.8542
5	412	TSAPCTITGTMGHF	0.92	0.9881
6	723	TPYELTPGATVPFL	0.858	0.9848
7	183	PEGYYNWHHGAVQY	0.745	0.9042
8	1052	WLKERGASLQHTAP	0.711	1.1089

**Table 2:** Discontinuous epitopes predicted via DISCO TOP 2.0 by using three-dimensional structure

Serial no	Position	Residue	Contact no.	Disco tope score
1	147	HIS	0	4.499
2	269	PRO	15	3.228
3	273	ASN	6	3.193
4	290	ASP	9	4.196
5	291	HIS	18	2.951
6	292	PRO	10	3.122
7	312	MET	0	3.904

**Fig. 2:**
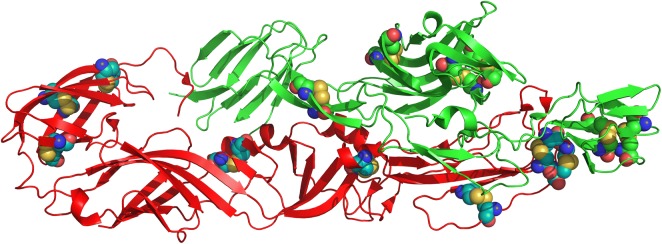
Site of predicted epitopes on chains of the crystal structure of CHIKV structural polyprotein highlighted through cartoon representation

To predict the beta turns in structural polyprotein, Chou and Fasman Beta turn analyzing algorithm measure surface exposed and hydrophilicity that play a critical role to initiate the immune response. Threshold of the tool was set at 0.988, it calculated the values which are 0.530 (minimum), 0.988 (average) and 1.443 (maximum). Graphical representation of Chou and Fasman’s output is shown in Fig. 1d. Results indicated that the regions from 60 to 100 amino acids and from 980 to 1020 amino acids are more inclined to induce B-turns in peptide structure. Experimental data has reported that those regions of epitope which interact with alleles or antibodies are mostly elastic in nature. To, detect the flexible regions of the target protein, Karplus and Schulz flexibility analyzing tool depicted that the region from amino acids from 60 to 100 sequence positions are highly flexible (Fig. 1e). Site of each predicted epitopes in its 3D structure is shown in Fig. 2.

### Recognition of T cell epitopes

T cell epitopes were predicted through the application of two different online servers. MHC class I alleles were predicted through ProPred while MHC class I alleles were predicted through Propred simply. Results of both MHC class I alleles and MHC class II alleles are given in Tables 3, 4. FASTA file of structural polyprotein sequences of the CHIKV was given as an input with 4% threshold level while keeping the proteasome filters and immune proteasome at on mode. Distinctive peptides against all MHC class I alleles were identified out of which we only retained peptides with antigenic properties. Antigenicity of ‘‘TAECKDKNL’’ peptide was higher amongst all peptides with the antigenicity score “1.6001” as shown italic in the Table 3. Similarly, for MHC class II alleles, sequence was also given in FASTA format to ProPred. Antigenicity score of ‘‘VRYKCNCGG’’ peptide predicted through Vaxijen 2.0 was 2.2127 which is greater (shown in italic) as compared to other MHC class II alleles binding peptides (Table 4).

**Table 3:** MHC class I peptides predicted through propred-I with their alleles and antigenicity scores

Serial no	Peptide	MHC class I alleles	Score
1	IFDNKGRVVAIVL	HLA-B*3501. HLA-B*3701, HLA-B*5101, HLA-B*5103, HLA-B*5201, HLA-B*51, HLA-Cw*0401, MHC-Kd	1.106
2	PLVPRNAEL	HLA-A2, HLA-A2.1, HLA-B8, HLA-Cw*0301	1.3876
3	VMHKKEVVL	HLA-A2, HLA-A*0201, HLA-A*0205, HLA-A3, HLA-A2.1, HLA-B*2702, HLA-B*2705, HLA-B*3902, HLA-B*5301, HLA-B*51, HLA-B7, HLA-B*0702, HLA-B8, HLA-Cw*0401, MHC-Dd	1.0207
4	LSVTLEPTL	HLA-A24, HLA-A2.1, HLA-B*3501, HLA-B*3902, HLA-B40, HLA-B*5301, HLA-B*51, HLA-B*5801, HLA-B60, HLA-B7, HLA-Cw*0301, HLA-Cw*0602, MHC-Db, MHC-Db revised, MHC-Kd, MHC-Ld	1.3989
5	*TAECKDKNL*	HLA-A1, HLA-B*3801, HLA-B*3902, HLA-B*5101, HLA-B*5102, HLA-B*5103, HLA-B*5801, HLA-B60, HLA-B8, HLA-Cw*0602, MHC-Dd	*1.6001*
6	GTLKIQVSL	HLA-A2, HLA-A*0205, HLA-B14, HLA-B*3701, HLA-B40, HLA-B*5801, HLA-B7, HLA-Cw*0301, HLA-Cw*0602, MHC-Db, MHC-Db revised, MHC-Kd	0.5303
7	LQISFSTAL	HLA-A2, HLA-A*0201, HLA-A*0205, HLA-A2.1, HLA-B14, HLA-B*2702, HLA-B*2705, HLA-B*3902. HLA-B40, HLA-B*5201, HLA-B*5301, HLA-B*51, HLA-B60, HLA-B62, HLA-B7, HLA-Cw*0301, HLA-Cw*0602, MHC-Db revised, MHC-Kd	0.6264

**Table 4:** T cell epitopes against MHC class II alleles through propred

Serial no	Peptide	MHC class II alleles	Score
1	FKRSSKYDL	DRB1_0305, DRB1_0309, DRB1_0701, DRB1_0703, DRB1_1114, DRB1_1120, DRB1_1302, DRB1_1323, DRB5_0101, DRB5_0105	1.054
2	LLANTTFPC	DRB1_0401, DRB1_0402, DRB1_0404, DRB1_0405, DRB1_0408, DRB1_0410, DRB1_0421, DRB1_0423, DRB1_0426, DRB1_1102, DRB1_1107, DRB1_1114, DRB1_1121, DRB1_1322, DRB1_1323	1.0945
3	LKIQVSLQI	DRB1_0101, DRB1_0402, DRB1_0404, DRB1_0405, DRB1_0408, DRB1_0410, DRB1_1101, DRB1_1102, DRB1_1104, DRB1_1106, DRB1_1114, DRB1_1120, DRB1_1121, DRB1_1128, DRB1_1301, DRB1_1302, DRB1_1305, DRB1_1311, DRB1_1321, DRB1_1322, DRB1_1323, DRB1_1327, DRB1_1328, DRB1_1501, DRB1_1502, DRB1_1506	0.9054
4	FVRTSAPCTI	DRB1_0101, DRB1_0102, DRB1_0421, DRB1_0426,	1.0082
5	VGFTDSRKI	DRB1_0402, DRB1_0701, DRB1_0703, DRB5_0101, DRB5_0105	1.0491
6	*VRYKCNCGG*	*DRB1_0404, DRB1_0405, DRB1_0410, DRB1_0423, DRB1_0801, DRB1_0802, DRB1_0804, DRB1_0806, DRB1_0813, DRB1_0817,*	*2.2127*
7	WVMHKKEVV	DRB1_0301, DRB1_0305, DRB1_0306, DRB1_0307, DRB1_0308, DRB1_0309, DRB1_0311, DRB1_0813, DRB1_1101, DRB1_1102, DRB1_1107, DRB1_1114, DRB1_1120, DRB1_1121, DRB1_1128, DRB1_1301, DRB1_1302, DRB1_1304, DRB1_1305, DRB1_1307, DRB1_1321, DRB1_1322, DRB1_1323, DRB1_1327, DRB1_1328, DRB1_1502	1.0706
8	LVVAVAALILIVVLCVSFSR	DRB1_0301, DRB1_0402, DRB1_1102, DRB1_1107, DRB1_1121, DRB1_1301, DRB1_1322, DRB1_1323, DRB1_1327, DRB1_1328, DRB5_0101, DRB5_0105	0.909

### Conservation analysis

**Fig. 3:**
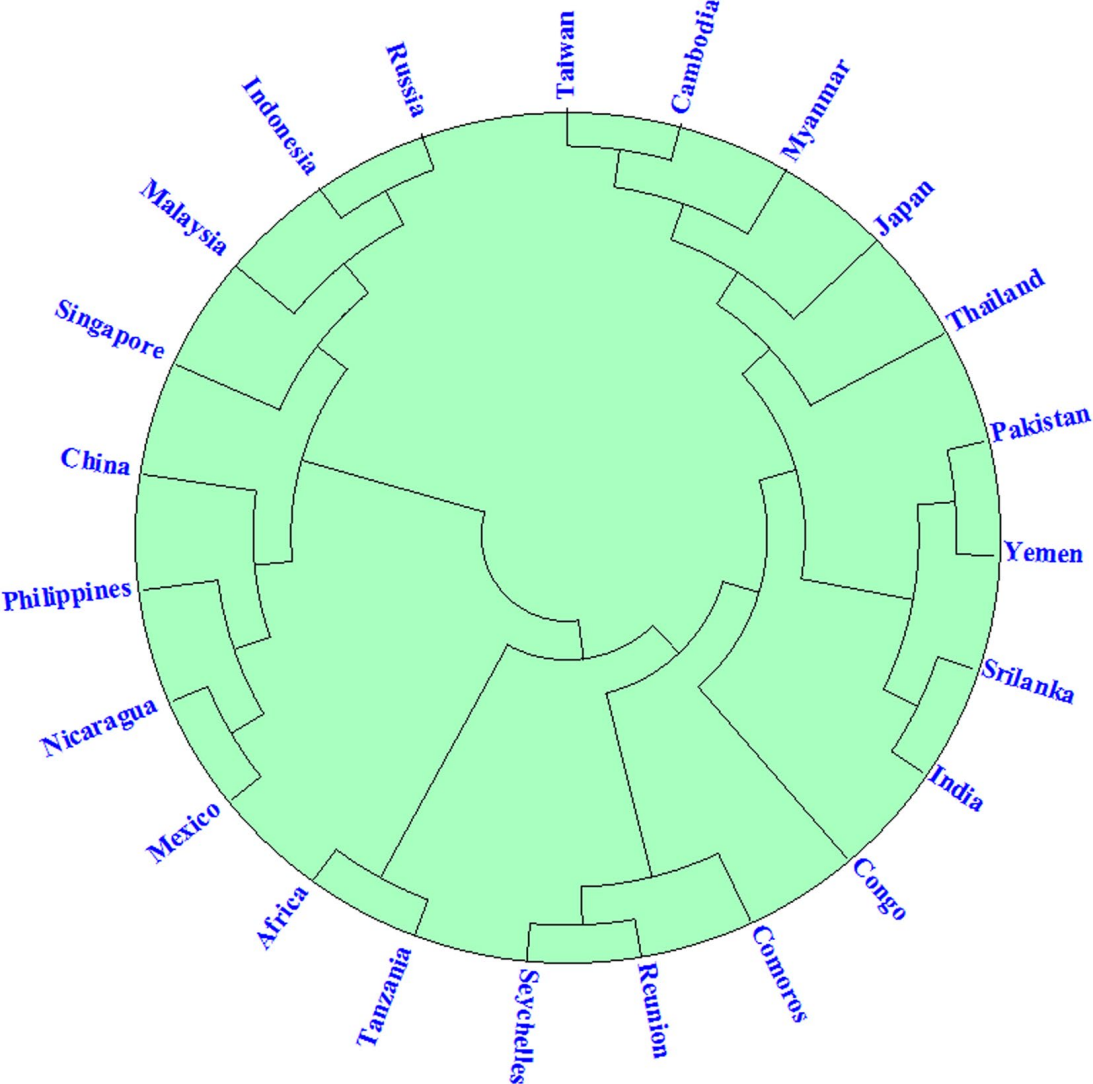
Phylogenetic tree illustrating the evolutionary conserveness of CHIKV strains form 23 countries

Sequence of CHIKV structural polyprotein isolates from 23 different countries was subjected to multiple-sequence alignment to analyze the conservation of selected epitopes through CLC workbench. It was observed that all the selected epitopes are highly conserved in all sequences used for analysis (Additional file 1: Figure S2). A phylogenetic tree was constructed to show the evolutionary relation of CHIKV belonging to 23 different countries as shown in Fig. 3. Evolutionary analysis revealed that south east Asian CHIKV strains including Pakistan and India stains are less divergent and more closely related to Congo and African CHIKV strains.

### Interaction study of predicted peptides with HLA alleles

Three dimensional structures of 4 MHC classes-I binding peptides were predicted through PEPFOLD. It produced five models of each peptide; one best model was selected for each peptide. Initially models were refined through energy minimization in MOE and peptide library comprised of four peptides was created to dock with solved structure of HLA-B7 allele.

**Fig. 4:**
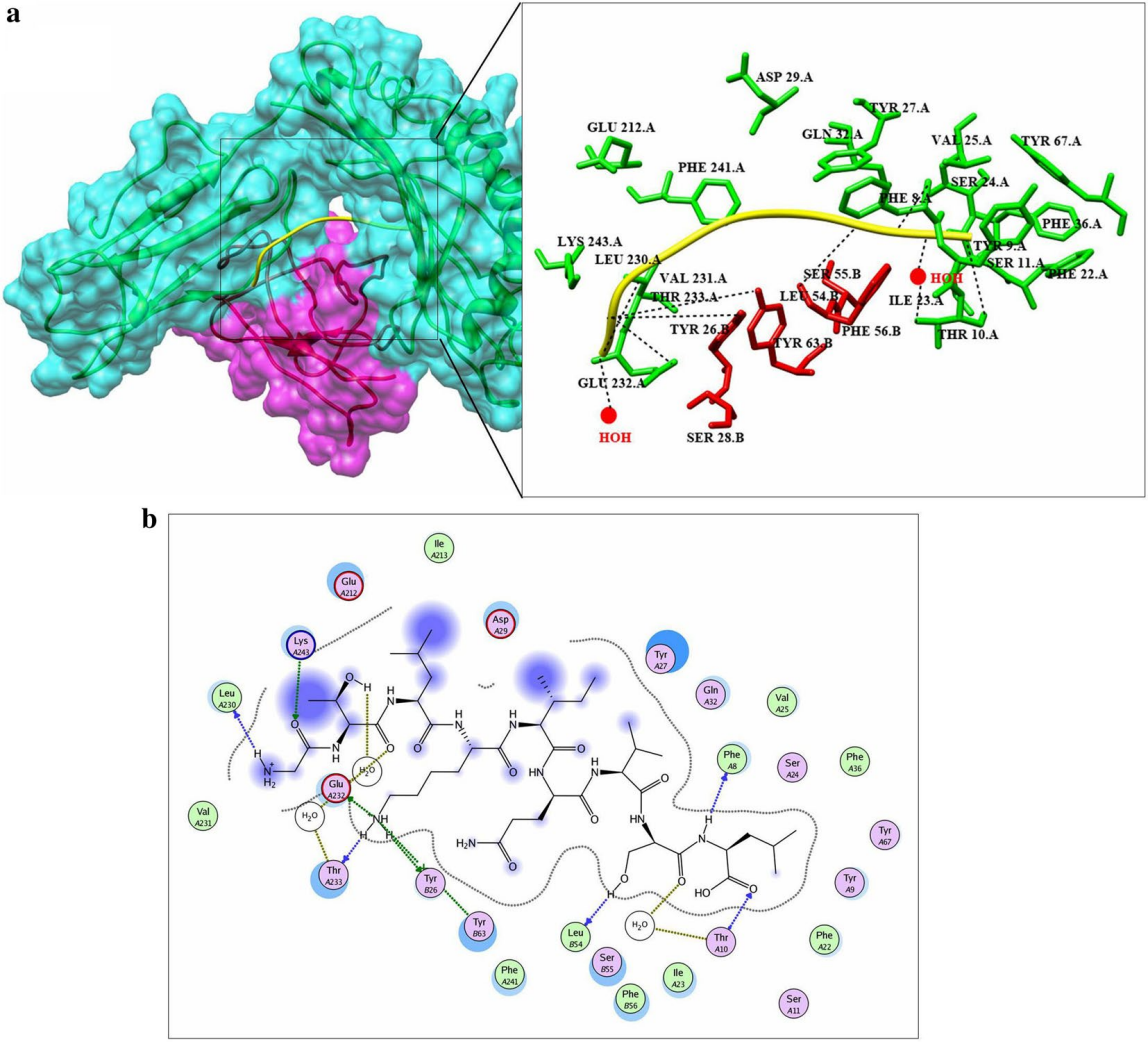
**a** Graphical representation of human HLA-B7 allele (shown in cyan and purple) with bound peptide 1 “VMHKKEVVL” (yellow). Multiple residues of the receptor protein (green) interact (dotted lines) with the peptide residues (red). **b** LigX interaction diagram illustrating the chain A and chains B residues interacting via hydrogen bond (green), two water mediated interaction (yellow), vanderval interaction (blue) to the peptide 1

**Fig. 5:**
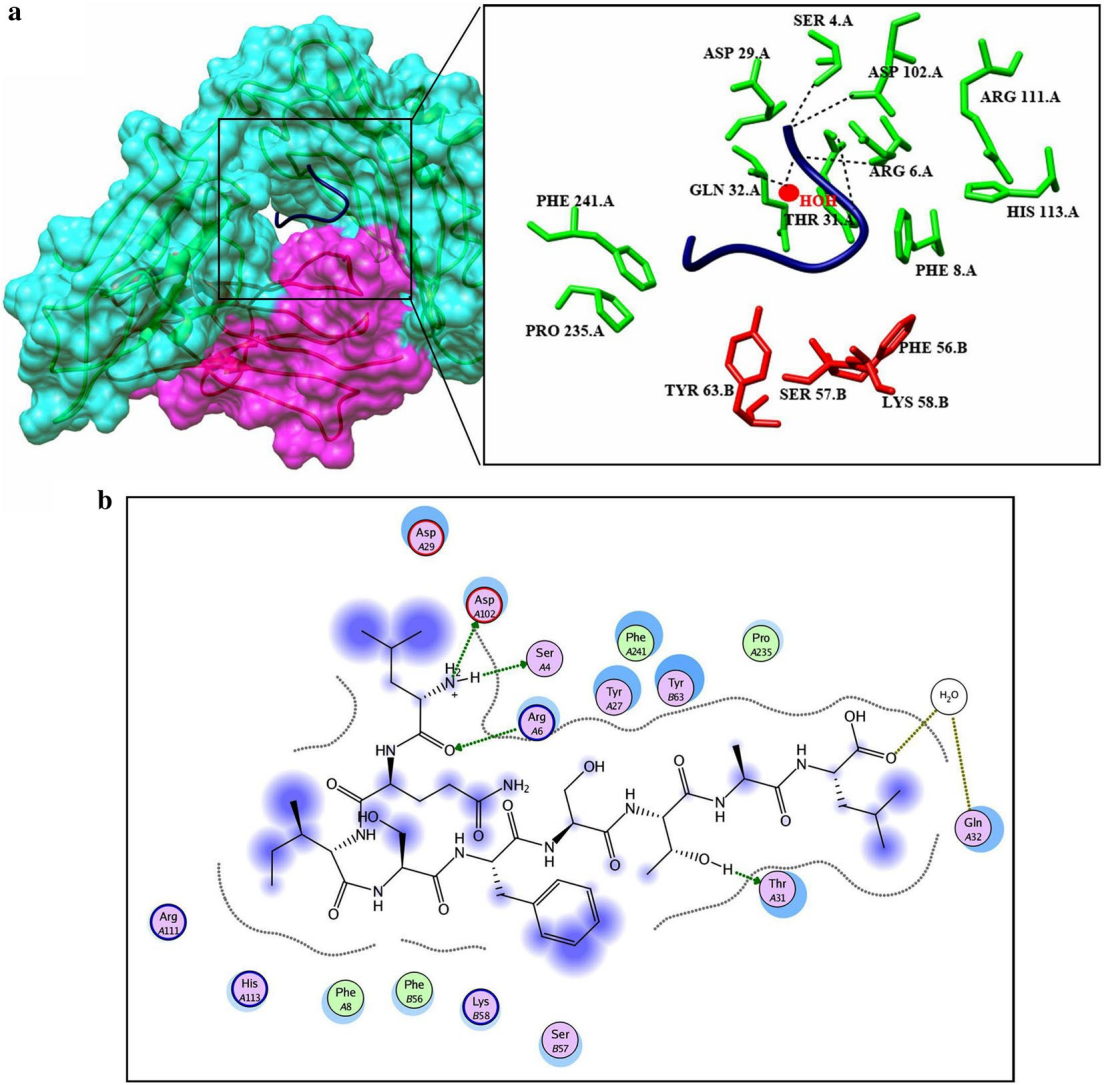
**a** Graphical representation of human HLA-B7 allele (shown in cyan and purple) with bound peptide 2 “GTLKIQVSL” (blue). Multiple residues of the receptor protein (green) interact (dotted lines) with the peptide residues (red). **b** LigX interaction diagram illustrating the chain A residues interacting via hydrogen bond (green), one water mediated interaction (yellow), vanderval interaction (blue) to the peptide 2

**Fig. 6:**
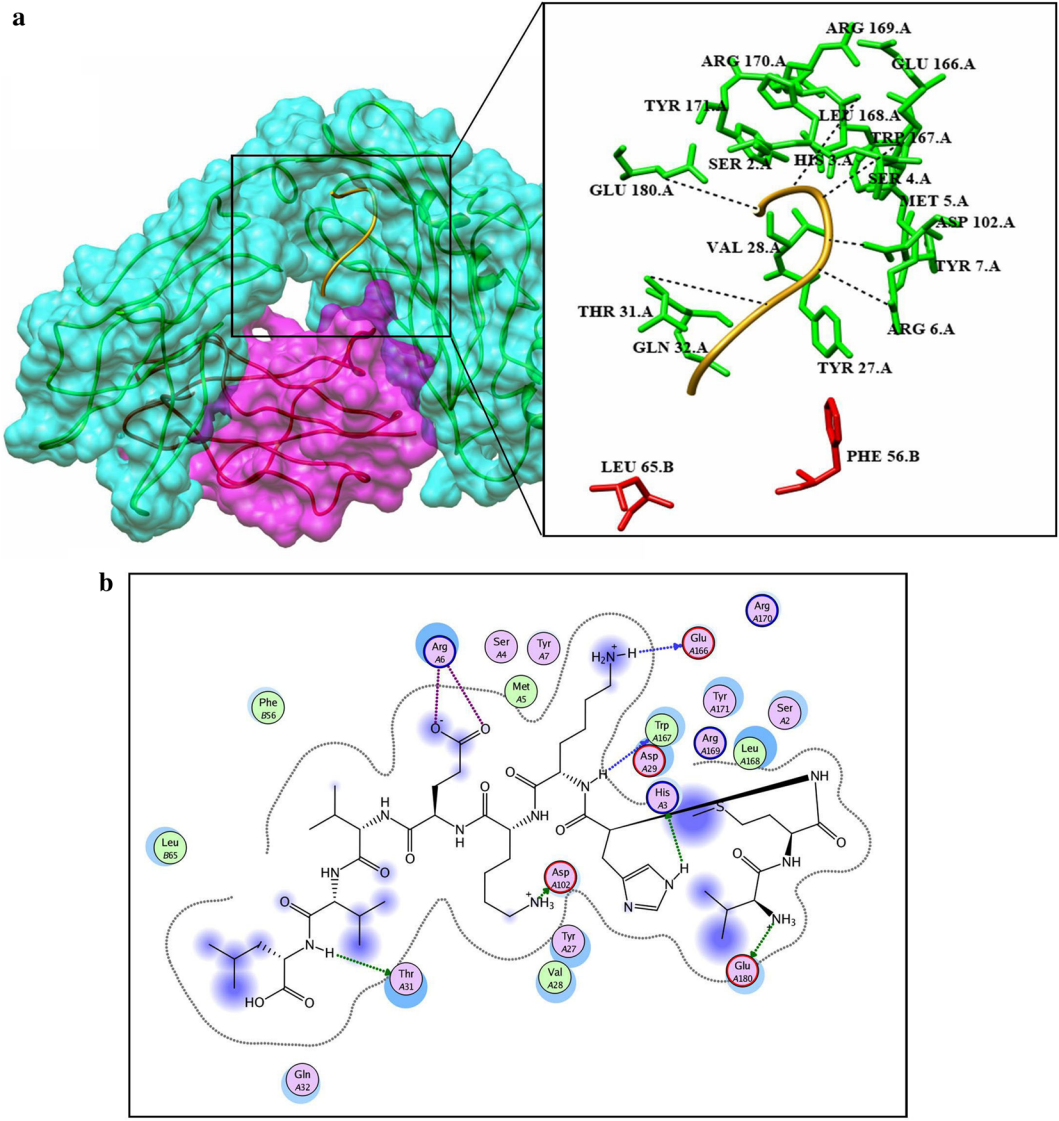
**a** Graphical representation of human HLA-B7 allele (shown in cyan and purple) with bound peptide 3 (yellow). Multiple residues of the receptor protein (green) interact (dotted lines) with the peptide residues (red). **b** LigX interaction diagram illustrating the chain A residues interacting via hydrogen bond (green) and vanderval interaction (blue) to the peptide 3

**Fig. 7:**
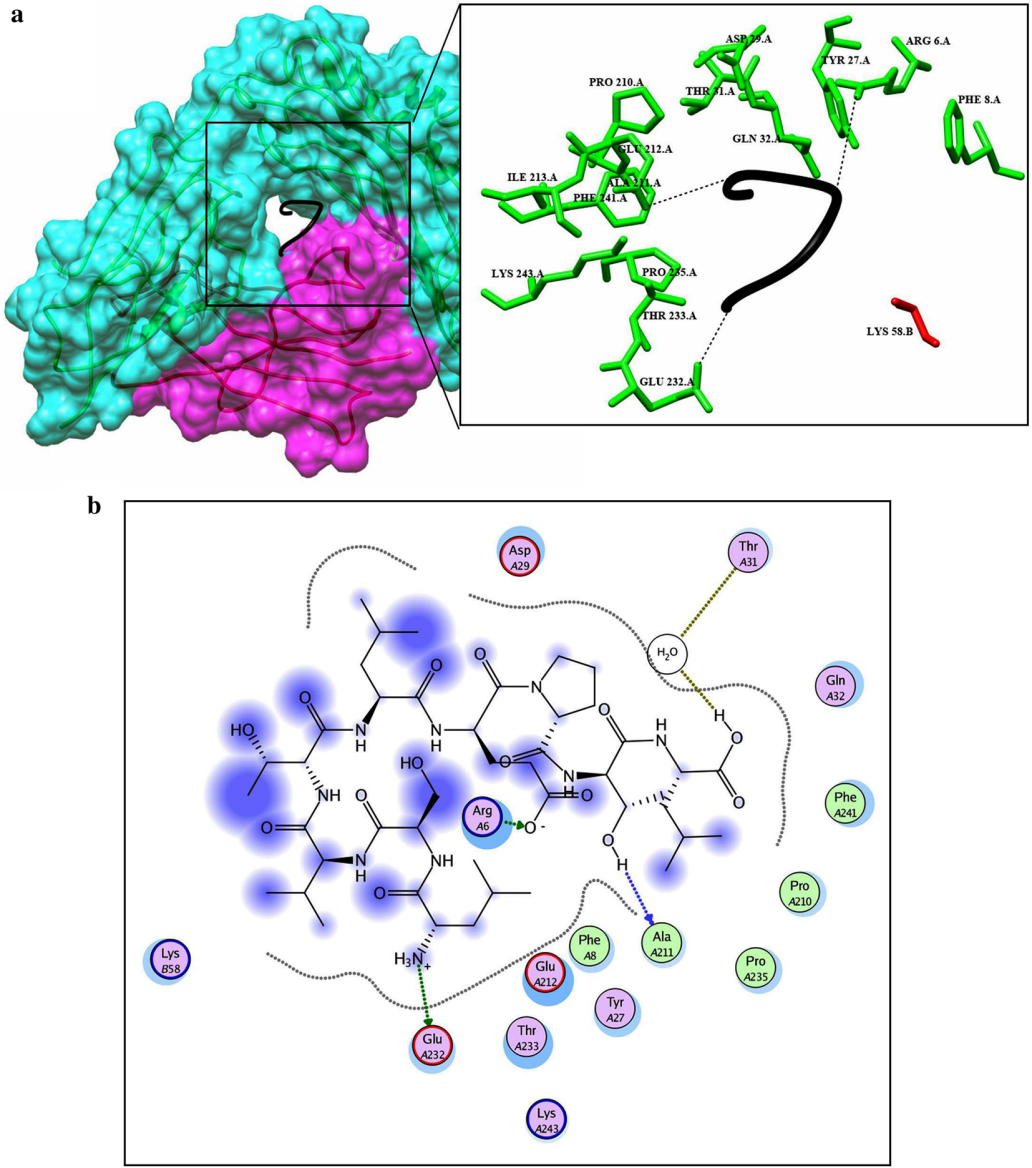
**a** Graphical representation of human HLA-B7 allele (shown in cyan and purple) with bound peptide 4 (black). Multiple residues of the receptor protein (green) interact (dotted lines) with the peptide residues (red). **b** LigX interaction diagram illustrating the chain A residues interacting via hydrogen bond (green) and vanderval interaction (blue) to the peptide 4

Before docking, energy minimization was performed for a library of four potential peptides and then docked against HLA-B7. Crystal structure of HLA-B7 (PDBID: 3VCL) was already available with co-crystallized peptide. So focused docking was performed by using same active pocket to dock our peptide library. Ten confirmations for each epitope were generated and top ranked conformations based on their dock scores were selected (Table 5). Later on, interaction analysis using ligX tool was done which revealed that the peptide “VMHKKEVVL” with highest dock score (− 20.2086 kcal/mol) is interacting with key catalytic residues of chain A. Human HLA-B7 is a hetero-dimer structure, from the interaction analysis it was revealed that Lys-243, Glu-232 while Tyr-26 and Tyr63 from B chains were making stable hydrogen bonds with the aforementioned peptide. In addition to this four water molecules were observed to be involved in water mediated interaction between the peptide and Thr-233 (A chain), and Thr-10 (B chain) shown in Fig. 4a, b. Peptide “GTLKIQVSL” was docked (dock score − 19.6688 kcal/mol) within the catalytic pocket of receptor protein through four hydrogen bond interaction with Ser-4, Arg-6, Thr-31 and Asp102 (chain A) and one water mediated interaction with Gln-32 (Fig. 5a, b). Peptide ranked 3th in Table 5 has − 17.3569 kcal/mol of dock score with three stable hydrogen bonds between peptide and His-3, Thr-31, Asp-102 and Glu-180 shown in Fig. 6a, b. Similarly, peptide ranked at 4th with dock score (− 14.5594 kcal/mol) got 2 stable hydrogen bond interactions with Arg-6 and Glu-232. While one water mediated interaction between peptide and receptor (Thr-31) can also be observed in Fig. 7a, b.

**Table 5:** Docking results predicted through molecular operating environment by using three-dimensional structure of peptides against HLA-B7

Serial no	Peptide	Docking score	Interacting residues
1	VMHKKEVVL	− 20.2086	Lys-243, Glu-232, Tyr-26, Tyr63, Thr-233, Thr-10
2	GTLKIQVSL	− 19.6688	Ser-4, Arg-6, Thr-31, Asp102, Gln-32
3	LSVTLEPTL	− 17.3569	His-3, Thr-31, Asp-102, Glu-180
4	LQISFSTAL	− 14.5594	Arg-6, Glu-232, Thr-31

### Allergenicity analysis

Allergenicity assessment of query protein was performed via Aller Hunter to predict whether it is allergenic or not based on the FAO/WHO standards. Results showed that our query sequence was non-allergenic with a score of 0.0 (SP = 89.3%, SE = 91.6%).

## Discussion

Emergence of new viral diseases in resource poor countries of Africa and Asia are adding a huge global disease burden. Chikungunya infection is not often recognized as a risk in developing countries and much less attention is paid towards the development of therapeutic solutions. Chikungunya infection mimics the symptoms of dengue and often co-infection of both the infection leads to misdiagnosis. In addition to this various mutation in CHIKV strains makes it resistant to already available antiviral molecules. There is no FDA approved reliable and specific drug against CHIKV infection is available in the market. Various recombinant vaccines have been designed which induces robust immune mechanisms in mice, but these vaccines are still in their clinical evaluation process. Pharmaceutical companies are neglecting CHIKV vaccine development as it is a lengthy as well as expensive procedure. To deal with the cost and time factor of the drug design phase, Chemo-informatics and immuno-informatics are of immense benefit. To develop cost effective and reliable therapeutic solutions, a detailed insight of human body immune response against viral agents is imperative. Advancements of immune-informatics tools have been instantly utilized by researchers to predict possible antigenic epitopes from CHIKV proteins (specially the structural polyprotein) for peptide based vaccine development [10]. CHIKV structural polyprotein plays a critical role in entry and fusion of CHIKV within human cells. Hence, to inhibit the passage of virus into the cell at the initial stages, structural polyprotein could be good target. In-silico approaches helped over in vitro experimental assays and tool in vaccine design specifically in terms of cost as well as time [35–37]. In current study, we have predicted different potential B&T cell antigenic determinants for structural polyprotein that can boost immune responses of the host.

In this study, computational approach was used to predict conserved epitope by performing multiple sequence analysis and epitope prediction tools. Once B and T cell epitopes were predicted their antigenicity, solvent accessibility, flexibility and disulphide bond potential was analyzed. Conservation of the predicted epitopes among CHIKV strains from different regions of world was analyzed to pick those epitopes that are specific to all. Conserved epitopes with high antigenicity score were selected to build their protein models using peptide building tools and docked with human HL-B7 allele to analyze enough binding specificity to induce immune response.

In epitope-based vaccine development a highly conserved epitope is expected to deliver a broader protection across different strains. Moreover, CHIKV being an RNA virus is more vulnerable to mutation due to a lack of proof reading activity of RNA polymerase. So, for reliable vaccine candidate, highly conserved epitope will ensure an effective and long-lasting immunity [19]. Online available tools like Propred I and Propred predicted T-cell antigenic determinants having attachment ability with alleles of MHC class-I and MHC class-II, In MHC class I “TAECKDKNL” and in MHC class II “VRYKCNCGG” epitopes interact with the highest number of HLA alleles and are greatly antigenic in nature. After conservation analysis it was found that as a result all predicted T-cell epitopes were conserved between CHIKV genomic sequences reported form 17 different countries. On the bias of docking score, binding potential with HLA-B7 and immunogenicity score, the peptides found in the present study may prove more immunogenic as compared to the earlier reported peptides. Predicted peptides might show the physicochemical instability, to overcome this limitation, several structural as well as physical modification strategies are available to enhance the poor physiochemical stability of peptides. These strategies are including peptidomimetic approach, prodrug approach, analogue formations, hydrophobic ion pairing, conjugation with fatty acids and use of substitute methods of drug administration [38]. Researchers have been working to gather data linked to CHIKV to understand its biology, transmission and patho-physiology in order to eliminate the disease completely. In the near future, we anticipate that predicted epitopes have therapeutic potential with an outstanding scope. Our immune-informatics examinations have proposed a strong T cell epitope along with a B cell epitope which will efficiently supports the development of potent peptide-based vaccines to deal with the CHIKV challenge.

## Conclusions

In recent studies novel antigenic epitopes of some essential and vital proteins revealed that can victoriously elicit response of immune system therefore becoming great peptide vaccines targets and protecting host from virus attack. So, the current research was conducted to predict antigenic epitopes of structural polyprotein of CHIKV. We carry out sequence, structure, and conservation analysis as well as homology modeling of structural polyprotein of CHIKV. This research discloses T and B cell epitopes which are antigenic and conserved among the CHIKV isolates of 17 different countries. These epitopes are capable to induce a particular immunologic response. We hope and rely that few of the antigenic epitopes proposed and investigated in this work might present a preliminary set of peptides for future vaccine development against CHIKV for control and prevention of this devastating epidemic.

## Additional file

**Additional file 1: Figure S1.** A) PSIPRED analysis of CHIKV structural polyprotein representing helix (tea-pink), stand (yellow) and coil region; B) Representation of asymmetric heterotrimeric complex showing A chain (blue), B chains (green) and F chains (red) of three dimensional (3D) structure of CHIKV structural polyprotein. C) cartoon representation of CHIKV structural polyprotein. **Figure S2.** Multiple sequence alignment illustrating the sequences consensus and conserveness of CHIKV strains form 23 countries. **Table S1.** Predicted disulfide bonds within GP. **Table S2.** Emini surface accessibility prediction results.

## Abbreviations

CHIKV: chikungunya virus
HLAs: human leukocyte antigens
MHC: major histocompatibility
