# Supplementary material for: Peptide vaccine against chikungunya virus: immuno-informatics combined with molecular docking approach
Source: J Transl Med. 2018 Oct 27;16:298. doi: 10.1186/s12967-018-1672-7 (PMC6204282; doi:10.1186/s12967-018-1672-7)
Supplement: Supplementary file 1 — Additional file 1: Figure S1. A) PSIPRED analysis of CHIKV structural polyprotein representing helix (tea-pink), stand (yellow) and coil region; B) Representation of asymmetric heterotrimeric complex showing A chain (blue), B chains (green) and F chains (red) of three dimensional (3D) structure of CHIKV structural polyprotein. C) cartoon representation of CHIKV structural polyprotein. Figure S2. Multiple sequence alignment illustrating the sequences consensus and conserveness of CHIKV strains form 23 countries. Table S1. Predicted disulfide bonds within GP. Table S2. Emini surface accessibility prediction results. [file 12967_2018_1672_MOESM1_ESM.docx]

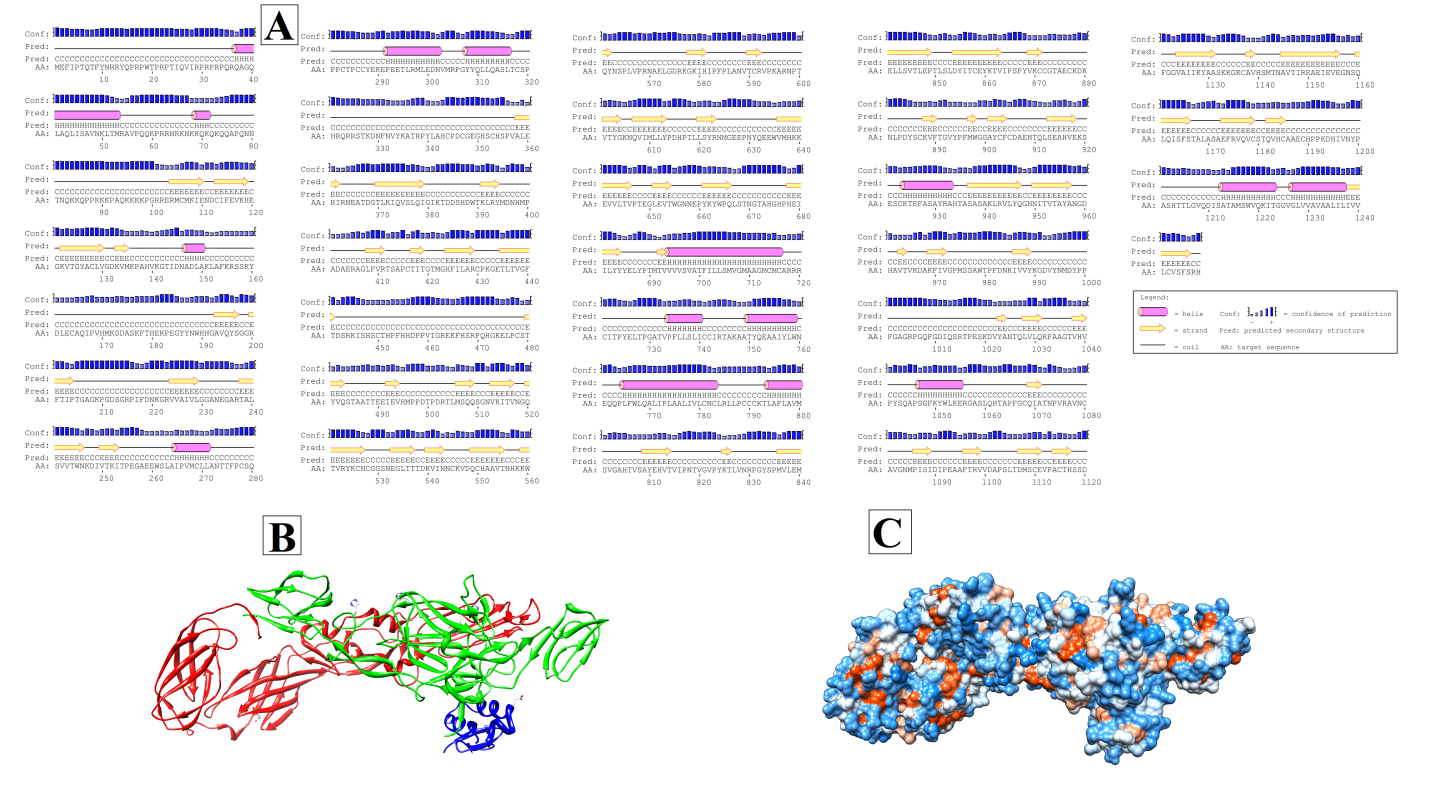


**Figure S1**: A) PSIPRED analysis of CHIKV structural polyprotein representing helix (tea-pink), stand (yellow) and coil region; B) Representation of asymmetric heterotrimeric complex showing A chain (blue), B chains (green) and F chains (red) of three dimensional (3D) structure of CHIKV structural polyprotein. C) cartoon representation of CHIKV structural polyprotein


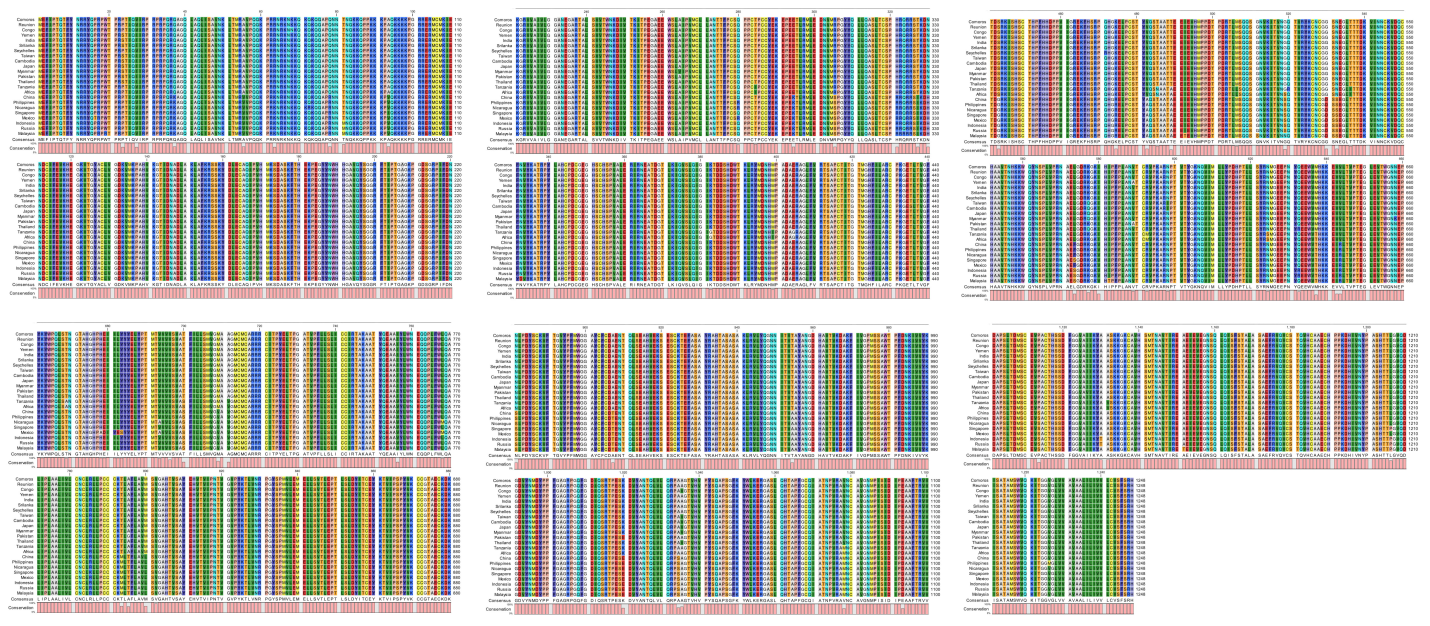


**Figure S2**: Multiple sequence alignment illustrating the sequences consensus and conserveness of CHIKV strains form 23 countries.

Table S1: Predicted disulfide bonds within GP

| Predicted Bonds |  | Score |
| --- | --- | --- |
| 106 - 416 | RRERMCMKIEN - RTSAPCTITGT | 0.99461 |
| 113 - 283 | KIENDCIFEVK - CSQPPCTPCCY | 0.99939 |
| 128 - 287 | VTGYACLVGDK - PCTPCCYEKEP | 0.98485 |
| 164 - 344 | KYDLECAQIPV - PYLAHCPDCGE | 0.66247 |
| 269 - 741 | AIPVMCLLANT - LLSLICCIRTA | 0.99668 |
| 278 - 591 | NTTFPCSQPPC - LANVTCRVPKA | 0.98807 |
| 286 - 887 | PPCTPCCYEKE - LPDYSCKVFTG | 0.97457 |
| 318 - 1068 | QASLTCSPHRQ - TAPFGCQIATN | 0.96092 |
| 347 - 790 | AHCPDCGEGHS - RLLPCCCKTLA | 0.98142 |
| 353 - 714 | GEGHSCHSPVA - MAAGMCMCARR | 0.73399 |
| 430 - 871 | FILARCPKGET - SPYVKCCGTAE | 0.99785 |
| 450 - 1115 | KISHSCTHPFH - CEVPACTHSSD | 0.99663 |
| 478 - 550 | GKELPCSTYVQ - CKVDQCHAAVT | 0.99436 |
| 528 - 1080 | RYKCNCGGSNE - VRAVNCAVGNM | 0.99957 |
| 545 - 789 | KVINNCKVDQC - LRLLPCCCKTL | 0.99711 |
| 716 - 1189 | AGMCMCARRRC - HCAAECHPPKD | 0.98898 |
| 721 - 781 | CARRRCITPYE - ALIVLCNCLRL | 0.93154 |
| 742 - 923 | LSLICCIRTAK - EKSESCKTEFA | 0.93965 |
| 783 - 903 | IVLCNCLRLLP - WGGAYCFCDAE | 0.98671 |
| 791 - 1242 | LLPCCCKTLAF - LIVVLCVSFSR | 0.99344 |
| 858 - 877 | LDYITCEYKTV - CGTAECKDKNL | 0.94159 |
| 872 - 1137 | PYVKCCGTAEC - SKKGKCAVHSM | 0.71482 |
| 905 - 1110 | GAYCFCDAENT - LTDMSCEVPAC | 0.82867 |
| 1179 - 1185 | FRVQVCSTQVH - STQVHCAAECH | 0.63053 |

Table S2: Emini Surface Accessibility Prediction Results

| Position | Residue | Start | End | Peptide | Score |
| --- | --- | --- | --- | --- | --- |
| 12 | **R** | 10 | 15 | YNRRYQ | 5.084 |
| 13 | **R** | 11 | 16 | NRRYQP | 5.017 |
| 14 | **Y** | 12 | 17 | RRYQPR | 6.11 |
| 31 | **R** | 29 | 34 | RPRPRP | 5.384 |
| 33 | **R** | 31 | 36 | RPRPQR | 6.03 |
| 34 | **P** | 32 | 37 | PRPQRQ | 5.331 |
| 59 | **Q** | 57 | 62 | PQQKPR | 5.444 |
| 60 | **K** | 58 | 63 | QQKPRR | 6.895 |
| 61 | **P** | 59 | 64 | QKPRRN | 6.403 |
| 62 | **R** | 60 | 65 | KPRRNR | 7.241 |
| 63 | **R** | 61 | 66 | PRRNRK | 7.241 |
| 64 | **N** | 62 | 67 | RRNRKN | 7.531 |
| 65 | **R** | 63 | 68 | RNRKNK | 7.689 |
| 66 | **K** | 64 | 69 | NRKNKK | 7.851 |
| 67 | **N** | 65 | 70 | RKNKKQ | 8.455 |
| 68 | **K** | 66 | 71 | KNKKQK | 8.633 |
| 69 | **K** | 67 | 72 | NKKQKQ | 7.476 |
| 70 | **Q** | 68 | 73 | KKQKQK | 9.297 |
| 71 | **K** | 69 | 74 | KQKQKQ | 8.051 |
| 72 | **Q** | 70 | 75 | QKQKQQ | 6.972 |
| 82 | **N** | 80 | 85 | NTNQKK | 5.01 |
| 83 | **Q** | 81 | 86 | TNQKKQ | 5.395 |
| 84 | **K** | 82 | 87 | NQKKQP | 5.781 |
| 85 | **K** | 83 | 88 | QKKQPP | 5.558 |
| 86 | **Q** | 84 | 89 | KKQPPK | 6.419 |
| 87 | **P** | 85 | 90 | KQPPKK | 6.419 |
| 88 | **P** | 86 | 91 | QPPKKK | 6.419 |
| 89 | **K** | 87 | 92 | PPKKKP | 5.731 |
| 95 | **K** | 93 | 98 | AQKKKK | 5.423 |
| 96 | **K** | 94 | 99 | QKKKKP | 8.301 |
| 291 | **E** | 289 | 294 | EKEPEE | 5.391 |
| 322 | **R** | 320 | 325 | PHRQRR | 5.306 |
